# Measurement and spatiotemporal evolution characteristics of dietary diversity among Chinese residents

**DOI:** 10.3389/fnut.2025.1480133

**Published:** 2025-01-27

**Authors:** Guangyuan Qin, Miaomiao Li, Shiwen Quan

**Affiliations:** ^1^School of Economics and Management, Beijing Forestry University, Beijing, China; ^2^Rural Development Institute, Chinese Academy of Social Sciences, Beijing, China

**Keywords:** dietary diversity, regional differences, spatiotemporal evolution, Dagum Gini coefficient, spatial Moran’s index

## Abstract

**Purpose:**

The purpose of this paper is to measure the dietary diversity and to analyze the regional characteristics, differences, and evolutionary trends of dietary diversity among Chinese residents.

**Methods:**

In this paper, the dietary diversity among Chinese residents was measured using the Shannon index based on the provincial-level food consumption data from 1995 to 2021. On this basis, the paper employs analysis methods such as kernel density estimation, spatial correlation test, and Dagum’s Gini coefficient to analyze the regional characteristics, differences, and trends of change in dietary diversity.

**Results:**

During the study period, the dietary diversity among Chinese residents showed an increasing trend. Among the four major geographic regions, the dietary diversity was highest in the southern region, followed by the northern region, northwest region, and Qinghai-Tibet region. Among the three major economic regions, the dietary diversity was highest in the eastern region, followed by the central region and western region. There was a significant positive spatial correlation in the dietary diversity among Chinese residents, and both high-high agglomeration and low-low agglomeration phenomena were strengthened. In terms of the trend of regional differences, whether overall differences, interregional differences, or intraregional differences, they all showed a shrinking trend. However, interregional differences were the main source of overall differences in dietary diversity among Chinese residents.

**Conclusion:**

The dietary diversity among Chinese residents shows an overall increasing trend, and there are regional differences in dietary diversity from the perspectives of economic and geographic regions, which have been narrowing over time.

## 1 Introduction

In recent years, with the acceleration of globalization and the improvement of living standards, there have been marked changes in people’s dietary status, both in terms of food types and food structure, reflecting the population’s demand for more nutritious and enriched diets. Dietary diversity has an important impact on individual health and nutritional status, and is often regarded as a necessary condition for a nutritious diet. On a global scale, enhancing dietary diversity is recognized as a key strategy for combating malnutrition and improving public health. For example, within the framework of the United Nations Sustainable Development Goals, dietary diversity is closely linked to food security and public health policies (1). Currently, countries around the world are facing challenges related to malnutrition, monotonous diets, and the burden of chronic diseases. This is particularly evident in developing countries, where changes in dietary patterns are occurring due to rising income levels, accelerating urbanization, and the shifting food supply dynamics brought about by globalization. As the most populous country in the world and the largest developing nation, studying the evolution of dietary diversity in China is not only crucial for understanding the health status of the country but also provides a representative case for the formulation of global nutrition security policies.

China’s vast territorial extent, along with its complex and diverse topography and climate, gives rise to significant regional disparities in agricultural production, which in turn have a profound impact on the dietary patterns of its residents. Moreover, as a developing country undergoing an economic transition, China faces many challenges common to other developing nations, such as economic imbalances and income inequality. In terms of diet, these economic disparities are reflected in the differences in food supply and consumption capacity. For instance, urban areas tend to have more abundant and diverse food supplies, which facilitate residents’ access to a wide range of foods, whereas economically underdeveloped regions often suffer from inadequate food supply conditions, and residents may find it difficult to afford more expensive food items. Theoretically, it follows that dietary diversity in China will exhibit significant geographical and economic disparities. This implies that policies aimed at improving dietary conditions and ensuring nutritional security must be regionally tailored. Specifically, in economically developed areas, policies such as providing nutritional subsidies to low-income groups could be effective, while in more remote regions, the focus should be on improving food supply infrastructure. Therefore, examining the regional characteristics and disparities of dietary diversity in China offers valuable insights into enhancing food supply conditions across different regions, thereby informing the development of targeted health promotion policies and nutrition improvement strategies. Furthermore, when international organizations formulate universal health standards or policies, it is essential to consider the diverse dietary needs of different countries and regions. Given the significant geographical and economic differences within China, the country serves as an important case study in global research on dietary diversity, offering critical lessons for the regionalization and customization of global health policies.

Since dietary diversity is closely related to nutritional health, it has always been one of the focal points in the field of nutrition. In the field of nutrition, research on dietary diversity has mainly focused on two aspects, one is research that explores the factors influencing dietary diversity in order to improve dietary diversity. These influencing factors include the income of the resident (2), the level of urbanization (3), the infrastructure (4), the diversity of crops or production (5, 6), etc.; and the second is the study of the link between dietary diversity and nutritional health status (7, 8).

Dietary diversity, which is also food consumption diversity, involves both food supply and consumption, and has increasingly become a key concern in the field of agricultural economics in the context of advocating food security. In the field of agricultural economics, in addition to studying the factors affecting dietary diversity, it also focuses on food security issues that are closely related to dietary diversity, and some of the literature focuses on linkage analysis, for example, Kerr et al. (9) explored in their study whether the adoption of agro-ecological farming methods by highly vulnerable households in sub-Saharan Africa can improve food security and dietary diversity; Bonuedi et al. (10) utilized Sierra Leonean data to analyze whether access to local food markets can mitigate seasonal fluctuations in household dietary diversity and food security. Another part of the literature examines food consumption diversity as a proxy for food security or nutritional security in the context of regional food security or nutritional security; Cheteni et al. (11) used the Household Dietary Diversity Score (HDDS) and the Household Food Consumption Score (HFCS) in their study to explore the consumption patterns of households and their food security status in the rural areas of the Eastern Cape Province of South Africa.

Relevant studies focusing on China are concentrated in the field of nutrition and are mainly concerned with the status of food consumption diversity among special populations and its relationship with nutritional status. For example, Alia et al. (12) used a micro-survey from residents of Huocheng County in the Ili Kazakh Autonomous Prefecture to obtain dietary diversity data and explored the relationship between dietary diversity and hypertension; Zhu et al. (13) used data from a cross-sectional survey of 10 cities in China to study the correlation between dietary diversity and self-reported incidence of illnesses and the number of illnesses in infants and young children. In contrast, in China’s agricultural economy, research on food tends to focus more on food supply and demand and the structure of food consumption (14), with less research on dietary diversity.

Taken together, despite there are a large number of empirical studies related to dietary diversity in different research fields, there are few studies on the dietary diversity of Chinese residents and regional differences, and they are usually based on different micro-survey samples with different research methodologies, which results in a lack of comparability of the measured dietary diversity of the residents, and fails to systematically reveal the changes in the national as well as provincial residents’ dietary diversity over the years and the regional differences. Therefore, the potential marginal contributions of this study lie in two areas: firstly, the use of provincial food consumption data to systematically measure the level of dietary diversity of the residents of China and the provinces from 1995 to 2021. The second is to analyze the regional characteristics, spatial and temporal evolution, and regional differences in the dietary diversity of Chinese residents using kernel density estimation and the Moran’s index.

## 2 Data and methodology

### 2.1 Data sources

In this paper, dietary diversity is measured using data on food consumption by residents of China at the national level and 31 provinces. The data span is 1995 to 2021. They are derived from the China Statistical Yearbook and the statistical yearbooks of provinces, municipalities and autonomous regions. Due to the inconsistency of the statistical caliber of the food consumption categories of residents in the provincial statistical yearbooks, and considering the availability of data, this study rearranges the raw food consumption data based on the statistical caliber of the food consumption categories of residents in the post-2013 statistical yearbooks, and categorizes them into 11 major categories: grains, vegetables, edible oils, pork, beef and mutton, poultry, eggs, aquatic products, milk, melons and fruits, and sugar, and finally obtained the per capita dietary data of the residents of China and each province. In order to further calculate the level of dietary diversity of the population, this study refers to the Chinese Food Composition Table (CFCT) to convert the consumption data of the population for the 11 food groups into calorie consumption data.

### 2.2 Measurement of dietary diversity

In studies of dietary diversity, there is no uniform specification for the use of diversity measures. A wide variety of dietary diversity indicators still exist in recent studies. Common indicators can be categorized into two types: one is counting indicators that only consider food types or groups, such as DDS, FVS (15, 16). These indicators are typically based on micro-level data, such as dietary data collected through the 24-h dietary recall methods; the other is indicators that consider both the types and distribution of food consumed by the population, representative indicators include Shannon Index and Simpson Index (17), which are also applicable to macro-level data.

Counting indicators (e.g., DDS) have the advantage of providing a concise picture of the dietary diversity of the resident; however, they are not effective in measuring dietary diversity when the data are relatively poorly disaggregated. For instance, food categories such as rice, meat, eggs, fruits and vegetables, and seafood may exhibit significant statistical differences at the individual or household level based on micro-level data. Yet, from a regional perspective, all provinces are likely to involve the consumption of these food categories, with the only distinction being the variation in consumption quantities. In such cases, the use of DDS to measure dietary diversity is largely ineffective. In contrast, the second group of indices, represented by the Shannon and Simpson indices, is more applicable. These indices include information on the distribution of food consumed along with the type of food, and are diversity indices with the connotation of homogeneity. Considering that the food categories of the macro-dietary data used in this paper are relatively abbreviated and mainly based on eleven food groups, the Shannon index^1^ is a better indicator for measuring the dietary diversity of the Chinese population than the counting index.

In measuring dietary diversity, the Shannon index is actually a weighted geometric mean of the relative abundance of various foods based on consumption. It can be expressed by the formula as follows:


$$\mathrm{Shannon}=-\overset{n}{\underset{i=1}{\sum }}s_{i}\ln s_{i}$$

where i represents a food group, n is the total number of food groups consumed by the population, and $s_{i}$ is the share of calories provided by food group i in the total calories per capita. The Shannon index takes the minimum value of 0 when only one type of food is consumed, and the maximum value of the Shannon index is obtained when the calories provided by various types of food are equal. In this paper, it is normalized to take values in the range of [0,1], with larger values indicating higher levels of dietary diversity.

### 2.3 Statistical analysis

#### 2.3.1 Kernel density estimation

In order to more intuitively judge the dynamic change characteristics of dietary diversity in China and different regions, this paper uses Stata18 to estimate the kernel density of the dietary diversity of residents in China and the regions. The kernel density curves of the dietary diversity of residents are generated by the following function:


$$f\left(x\right)=\frac{1}{nh}\overset{n}{\underset{i=1}{\sum }}K\left(\frac{x_{i}-x}{h}\right)$$

Where n is the number of regional observations, and $x_{i}$ denotes the observed value, andx is the mean of the observations; K(∙) represents the kernel density function, and h represents the window width of kernel density estimation. Commonly used kernel density functions are Gaussian kernel, Epanechnikov kernel, etc. This paper is based on the Gaussian kernel function to discuss.

#### 2.3.2 Moran’s I

In this paper, we used stata18 to calculate the Moran’s index to investigate the spatial correlation and spatial evolution characteristics of the dietary diversity of residents in 31 provinces and China. The Moran’s I index is one of the commonly used methods for measuring spatial autocorrelation in spatial data (18–22). Its values range from [−1, 1], with positive values indicating spatial positive correlation, meaning that observations in neighboring regions tend to be similar; negative values indicating spatial negative correlation, meaning that observations in neighboring regions tend to differ, reflecting a dispersed pattern; and a value of 0 indicating no spatial correlation. The global Moran’s index is used to reflect the overall spatial correlation of the dietary diversity of Chinese residents, while the local Moran index is used to examine the spatial aggregation of the dietary diversity of residents in localized areas. The formula for calculating the global Moran index is as follows:


$$\mathrm{Mora}n^{\prime }s\;I=\frac{\sum_{i=1}^{n}\sum_{j=1}^{n}W_{ij}\left(Y_{i}-\bar{Y}\right)\left(Y_{j}-\bar{Y}\right)}{S^{2}\sum_{i=1}^{n}\sum_{j=1}^{n}W_{ij}}$$

In this study, we used two different spatial weight matrices, the spatial contiguity matrix and the economic distance matrix, to calculate the Moran’s I index. The spatial contiguity matrix is used to examine the geographic spatial autocorrelation of dietary diversity among residents. This is because neighboring provinces and municipalities are likely to share similar agricultural production conditions, with shorter transportation distances and more frequent trade and food mobility, which may result in more similar market supply structures and dietary cultures in adjacent regions. Therefore, dietary diversity may exhibit spatial clustering or similar trends of change. The spatial contiguity matrix is constructed based on whether provinces are geographically adjacent; if two provinces are adjacent, the weight is set to 1, otherwise, it is set to 0. The diagonal elements are set to 0.

Given that economic factors are an important determinant of food availability, this study also uses the economic distance matrix as one of the spatial weight matrices. The economic distance matrix more accurately reflects the impact of economic interactions between provinces and municipalities on the spatial distribution of dietary diversity. The economic distance weight matrix is based on the economic differences between provinces and municipalities, with the matrix elements defined as the reciprocal of the absolute difference in per capita GDP between two provinces.

#### 2.3.3 Dagum’s Gini coefficient and its decomposition

The Dagum Gini coefficient (23) and its decomposition is one of the commonly used methods in the study of regional differences. It can decompose the overall Gini coefficient into intra-group coefficient, inter-group coefficient and hypervariance density, in which the intra-group coefficient reflects the disparity of the level within each region; the inter-group coefficient reflects the disparity of the level between regions; and the hypervariance density reflects the phenomenon of overlap among the regions, which reflects the relative disparity situation. Therefore, Dagum Gini coefficient can better identify the source of regional disparities. This paper uses Matlab software to measure the Dagum Gini coefficient to explain the composition and sources of regional differences in dietary diversity levels.

## 3 Results

### 3.1 Overall situation and trends of dietary diversity among Chinese residents

**Figure 1:**
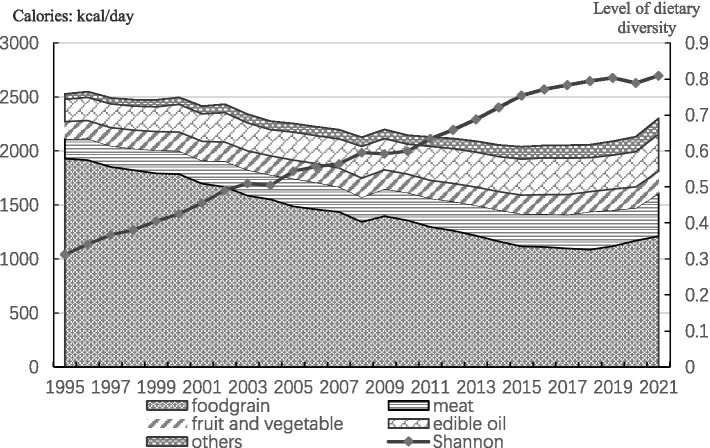
Trends in dietary diversity and dietary structure of the Chinese population, 1995–2021.

Figure 1 depicts the changes in the dietary structure and diversity level of Chinese residents from 1995 to 2021, where the dietary diversity level of Chinese residents is calculated from the national per capita dietary data. In the figure, the sum of the calorie shares of each food category is the total calories provided by the eleven food categories consumed by Chinese residents in that year, with meat indicating the sum of pork, beef, sheep, and poultry, and the other summarized food categories consumed by residents in lesser quantities, including milk, eggs, aquatic products, and sugar. From the point of view of the total per capita caloric intake of residents, since 1995, the total caloric intake of Chinese residents has shown a decreasing trend. Between 1995 and 2019, the total per capita caloric intake of residents dropped from more than 2,500 kcal to about 2,000 kcal, with a decline close to 1/5 of the original caloric intake; the level of dietary diversity has also shown a steady rise, and by 2019, the level of Chinese residents’ dietary diversity has risen to about 2.6 times that of 1995. Since 2019, the per capita caloric intake of residents has instead risen due to the impact of the New Crown Pneumonia epidemic, and the change in the level of dietary diversity of residents has been reversed.

According to Bennett’s law of food consumption, as the economy develops and incomes grow, people’s consumption of starchy staple foods such as rice and flour gradually decreases, while their consumption of nutrient-rich meats, vegetables and fruits increases substantially. According to Figure 1, it can be seen that the adjustment of the dietary structure of Chinese residents is in line with Bennett’s law. First, in the dietary structure of Chinese residents, the decline in the share of calories provided by food is very significant, from 76.3% in 1995 to 52.7% in 2021. On the other hand, the share of calories provided by other food groups, such as meat and fruits and vegetables, has been increasing, especially for meat and edible oil, which have risen from 7.2 and 8.2% to 17.3 and 14.9%, respectively. Overall, the evenness of Chinese residents’ dietary consumption has risen since 1995, which is a reflection of the increased level of dietary diversity. At the same time, the nutritional status of Chinese residents has improved significantly. Relevant data^2^ indicate that the malnutrition rate among Chinese adults was 6.0% in 2015, a decrease of 2.5% compared to 2002. The issue of stunted growth among rural children has been fundamentally addressed, with the stunting rate for children under the age of six in rural areas decreasing from 11.3% in 2015 to 5.8% in 2020. Similarly, the stunting rate for children and adolescents aged 6–17 years decreased from 4.7 to 2.2%.

It is important to note that while the improvement in dietary diversity has contributed to the enhancement of residents’ nutritional status, issues related to an imbalanced dietary structure persist. As shown in the figure above, the proportion of energy from fats in the diet of Chinese residents has continued to rise. The “China National Nutrition and Chronic Disease Report (2020)” indicates that the intake of edible oils and salt by Chinese residents is far above the recommended levels, while the consumption of fruits, beans and soybean products, and dairy products remains insufficient. The overweight and obesity rates among residents of all age groups in both urban and rural areas continue to increase. By 2020, more than half of the adult population was overweight or obese, and the overweight and obesity rates for children and adolescents aged 6–17 years and those under 6 years were 19 and 10.4%, respectively. Therefore, when considering improving nutritional status through dietary diversification, it is crucial to also emphasize the guidance of a healthy dietary structure.

### 3.2 Regional characteristics of dietary diversity of Chinese residents

**Figure 2:**
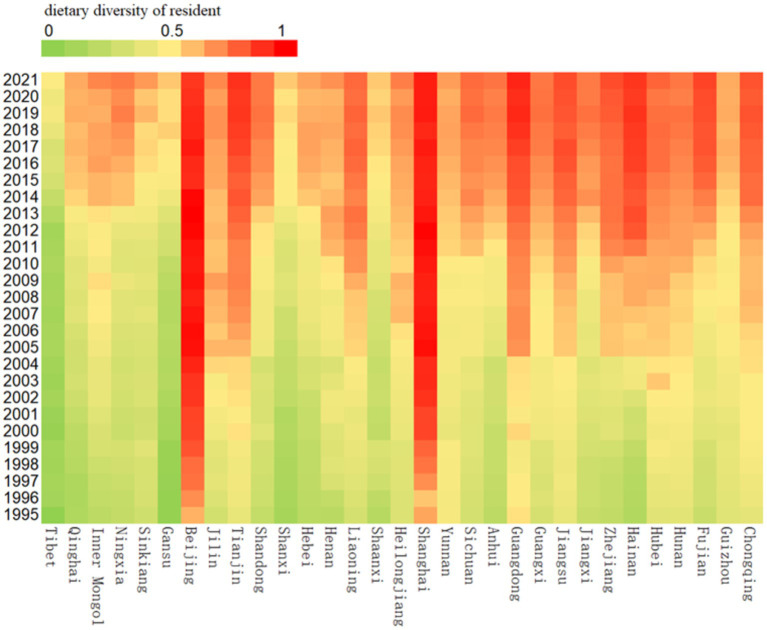
Dietary diversity levels of residents in 31 provinces and cities in China, 1995–2021.

China has a vast geographic area and diverse topographic and climatic conditions. Under the combined effect of natural geographic conditions and traditional culture, the diets of residents have obvious regional differences. In the study of Qin et al. (24), the dietary structure characteristics of four major geographic regions, namely, northwest, north, south, and Qinghai-Tibet, were summarized; while Wang et al. (25) investigated the regional evolutionary characteristics of the dietary structure of the Chinese population at multiple scales, including three major regions in the east, central, and west, nine major agricultural regions, and provincial units. In order to explore the regional characteristics of dietary diversity of Chinese residents, this study also needs to partition the 31 provinces and cities in the sample. Figure 2 depicts the levels of dietary diversity in 31 provinces and cities in China (excluding Taiwan Province) from 1995 to 2021. According to the figure, the level of dietary diversity of residents in each province and city shows considerable differences, but in general, provinces and cities with a high level of economic development and diversified production conditions have a higher level of dietary diversity of residents. Considering the regional characteristics of dietary structure and the data characteristics of residents’ dietary diversity presented in Figure 2, this study will divide the regions from two perspectives: on the one hand, considering the natural geographic distribution, the 31 provinces and municipalities in the sample will be divided into four major regions, namely, the north, the south, the northwest, and the Qinghai-Tibet, and on the other hand, considering the state of economic development, the 31 provinces and municipalities will be divided into three major regions, namely, the east, the center, and the west (The specific divisional criteria are shown in Supplementary Table S1).

**Table 1:** Global Moran’s index of the level of dietary diversity of the population, 1996–2021.

Year	Based on the spatial adjacency matrix		Based on the economic distance matrix	
	Moran’s I	*P*_value	Moran’s I	*P*_value
1996	0.1553	0.111	0.2870	0.001
1997	0.1039	0.243	0.3377	0.000
1998	0.0884	0.298	0.3711	0.000
1999	0.0739	0.356	0.4128	0.000
2000	0.2308	0.023	0.4377	0.000
2001	0.2014	0.043	0.4114	0.000
2002	0.1965	0.047	0.4341	0.000
2003	0.2406	0.018	0.4242	0.000
2004	0.2767	0.008	0.4014	0.000
2005	0.3600	0.001	0.3804	0.000
2006	0.3582	0.001	0.3867	0.000
2007	0.3456	0.001	0.3279	0.000
2008	0.3877	0.000	0.3181	0.000
2009	0.3784	0.000	0.3374	0.000
2010	0.4041	0.000	0.3553	0.000
2011	0.3659	0.001	0.3066	0.000
2012	0.358	0.001	0.3039	0.000
2013	0.3668	0.001	0.327	0.000
2014	0.3688	0.000	0.3566	0.000
2015	0.4279	0.000	0.3462	0.000
2016	0.4082	0.000	0.3534	0.000
2017	0.4099	0.000	0.3423	0.000
2018	0.4645	0.000	0.3319	0.000
2019	0.4348	0.000	0.3646	0.000
2020	0.4583	0.000	0.3879	0.000
2021	0.4166	0.000	0.4007	0.000

#### 3.2.1 Time-evolving kernel density-based characterization

**Figure 3:**
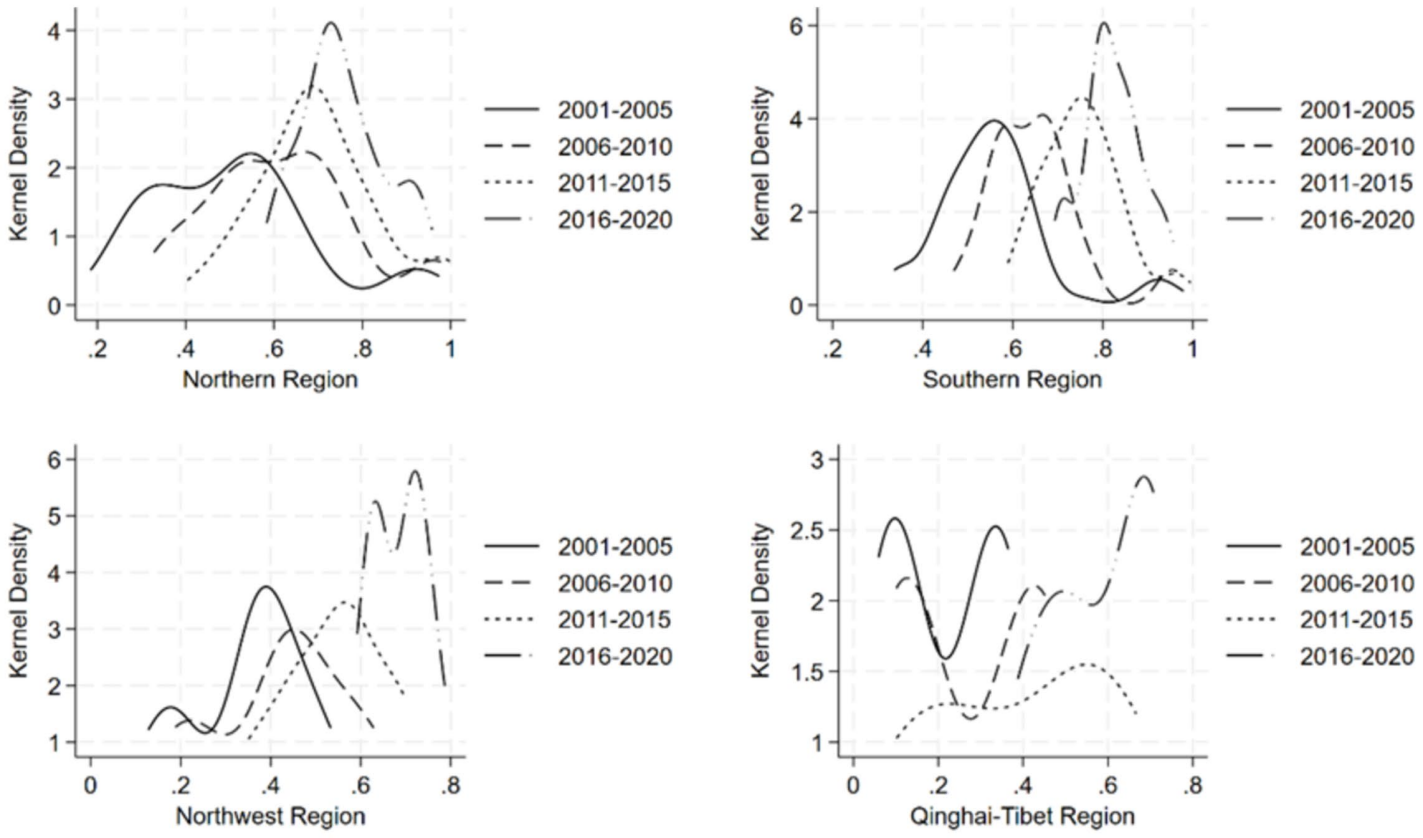
Kernel density map of dietary diversity of the population in the north, south, northwest, and Qinghai-Tibet regions.

In this paper, the temporal evolution of the distribution of dietary diversity of the Chinese population is characterized by the kernel density estimation method. Figure 3 depicts the multi-year kernel density curves of dietary diversity in the four major regions according to their physical geography. In terms of the direction of movement of the center of the kernel density curve, all the four regions show a rightward trend, indicating that the level of dietary diversity of the residents in the four regions has been increasing during the sample period. In terms of the distribution pattern of the peaks of the kernel density curves, the width of the peaks in the four regions shows a narrowing trend, indicating that the degree of dispersion of the dietary diversity of the residents in the four regions has narrowed. In terms of the number of peaks, the number of peaks in the northern region changed from double peaks to single peaks, and the phenomenon of multipolar distribution of dietary diversity disappeared. The southern region is dominated by single peaks, and there is no obvious polarization phenomenon. While the Northwest and Qinghai-Tibet regions have more obvious bimodal peaks, by 2016–2020, the bimodal peaks in the Northwest region are closer together, while the two wave peaks in the Qinghai-Tibet region have obvious differences in height, indicating that the level of dietary diversity of the residents within the two regions is not balanced but the phenomenon of imbalance over time has weakened. In terms of the extensibility of the distribution of the kernel density curves, both the northern and southern regions are characterized by right-sided trailing, and the trend has been weakened over time, suggesting that there is a gap between the northern and southern provinces due to the existence of a small number of residents with high levels of dietary diversity, but the gap has been narrowed to a certain extent in recent years. In the Northwest, there is a left-sided trailing feature that gradually disappears, indicating that there is a gap between the provinces in the Northwest due to the existence of a few low-level inhabitants’ dietary diversity and that the gap has narrowed over time.

**Figure 4:**
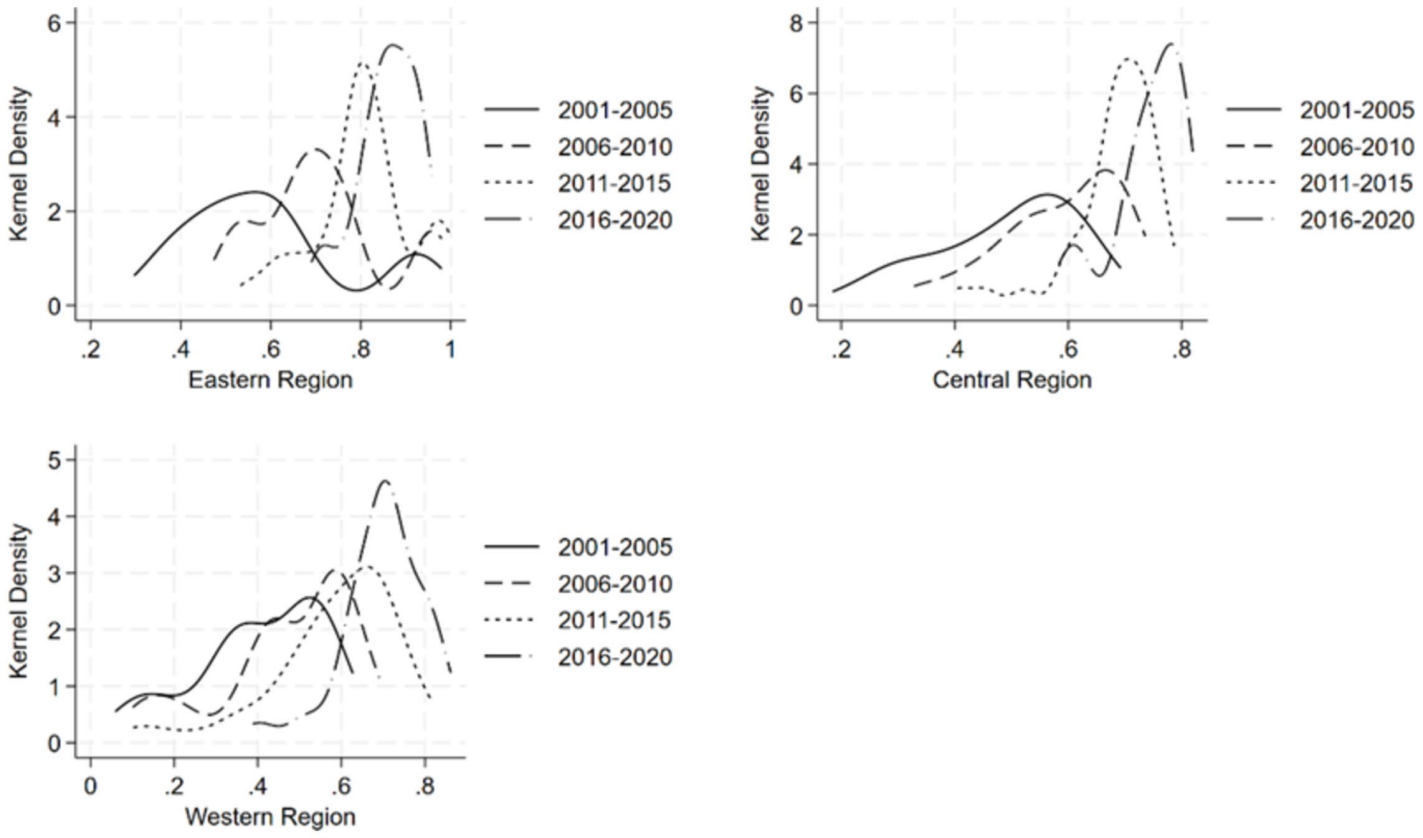
Kernel density map of dietary diversity of the population in eastern, central, and western regions.

Figure 4 depicts a multi-year kernel density plot of dietary diversity in the three major regions of the East, Central and West. It is clear that, consistent with the four major geographic regions, there is an upward trend in the level of dietary diversity of the residents of the three major regions of the East, Central and West, which are categorized according to their economic development status. In terms of the distribution pattern of the peaks of the kernel density curve, the width of the peaks in the East, Central and West regions has been narrowing, indicating that the degree of dispersion of the dietary diversity of the residents of the three regions has been narrowing. In terms of the number of peaks, during the period of 2001–2010, the dietary diversity of the residents in the eastern region was obviously multi-polarized, and the side with lower levels of dietary diversity was dominant, while the multi-polar distribution of the residents’ dietary diversity disappeared later, indicating that the level of dietary diversity of the residents in the eastern region was more balanced. The central region is dominated by a single peak, and there is no obvious polarization phenomenon. The western region also changed from bimodal to unimodal, but the difference between the bimodal peaks was not very significant, so the internal imbalance in the level of dietary diversity of the residents was not serious compared with the eastern region. In terms of the extensibility of the distribution of the kernel density curves, the three regions as a whole show a trailing left-hand side and a weakening trend over time, suggesting that there are gaps within the three regions due to the dietary diversity of a small number of residents with low dietary diversity and that the gaps are narrowing over time.

It is evident that differences in natural geographical conditions and economic factors have a significant impact on the variations in dietary diversity among residents. From the perspective of natural geographic divisions, the southern region exhibits higher dietary diversity, followed by the northern and northwestern regions, and finally the Qinghai-Tibet region, which generally aligns with empirical expectations. This is due to, on the one hand, the fact that this pattern is consistent with the diversity of agricultural production in China. According to a study by Zeng et al. (26) on the diversity of agricultural crops in China, the southern region indeed shows higher crop diversity, while the Qinghai-Tibet region has relatively lower crop diversity. On the other hand, natural geographic conditions themselves have a profound impact on economic development and infrastructure construction. The data show that during the study period, the per capita GDP across the four major geographic regions ranked from high to low as follows: southern, northern, northwestern, and Qinghai-Tibet region.

This pattern is also reflected in the three major regions based on economic conditions—eastern, central, and western. The eastern region has a higher level of economic development and relatively greater agricultural production diversity, while the western region shows the opposite. It is noteworthy that, compared to the eastern region, the central region exhibits higher agricultural production diversity, yet its dietary diversity level is lower. This suggests that economic conditions compensate for the lack of agricultural diversity. During the study period, the per capita GDP of the eastern region was 2.44 times higher than that of the central region; although the economic gap has narrowed in recent years, by 2021, the per capita GDP of the eastern region was still 1.7 times that of the central region.

#### 3.2.2 Characterization of the spatial evolution based on the Moran’s index

In order to explore the spatial correlation of dietary diversity of Chinese residents, this paper uses Moran’s I (Moran’s index) for analysis. Firstly, the spatial correlation test of global Moran’s I based on the spatial adjacency matrix and economic distance matrix respectively, and the detailed results are shown in the following Table 1. The results show that global Moran’s I based on the spatial adjacency matrix is always positive, and in general, it has become larger over time, and it is not significant at the beginning of the sample, and has been significant at the 5% level since 2000; global Moran’s I based on the economic distance matrix is also positive, but there is no obvious pattern of change in the value and it is always significant at the 1% level. Obviously, the spatial correlation of the dietary diversity of Chinese residents is significantly positive, and the spatial correlation of neighboring provinces and cities gradually increases over time, indicating that the dietary diversity of the residents of a certain province or city is more and more strongly influenced by the dietary diversity of the residents of neighboring provinces and cities. Therefore, in order to improve the dietary diversity of the residents of a region, we can consider starting from the region, and drive the development of the dietary diversity of the residents of the region through the neighboring regions.

**Figure 5:**
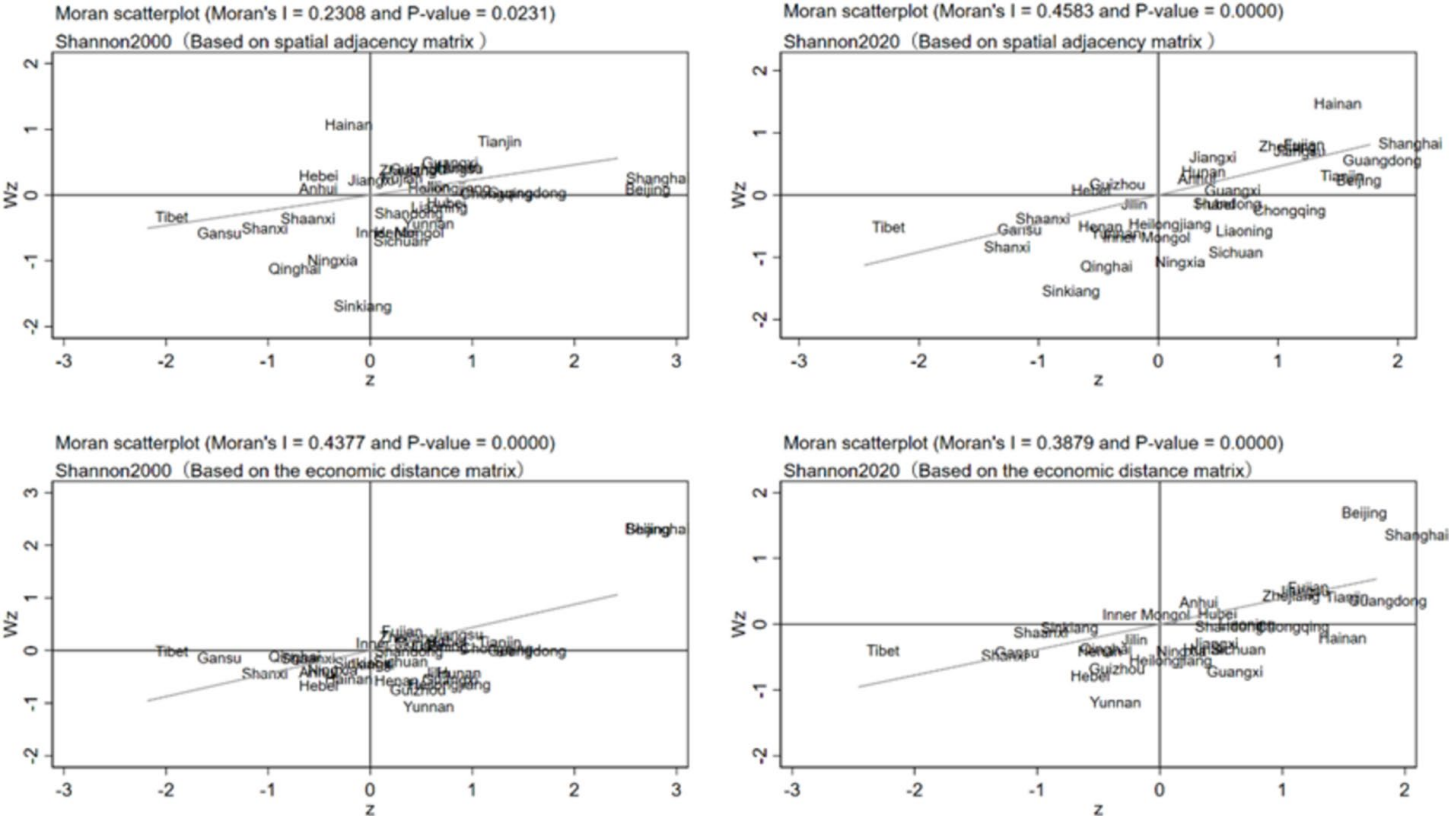
Scatter plot of Moran’s I index of dietary diversity of the population.

Further, this paper observes the spatial correlation of dietary diversity of residents in 31 provinces and cities through localized Moran’s I index. Figure 5 depicts the distribution of Moran’s I index of dietary diversity in 31 provinces and cities in China in 2000 and 2020 based on spatial adjacency matrix and economic distance matrix. The scatter plot of Moran’s index based on the spatial adjacency matrix shows that most of the provinces and cities are distributed in the first and third quadrants, indicating that the dietary diversity levels of the 31 provinces and cities have obvious spatial clustering, confirming the results based on the global Moran’s I index. Provinces and cities with high levels of dietary diversity are mainly concentrated in the first quadrant, including Beijing, Tianjin, Shanghai and other provinces and cities, indicating that these provinces and cities also have high levels of dietary diversity in neighboring provinces and cities; while provinces and cities in the northwest and Qinghai-Tibet regions, which have low levels of dietary diversity, are distributed in the third quadrant, and their neighboring provinces and cities also have low levels of dietary diversity. Comparing the distribution of Moran’s I index between 2000 and 2020, there is an increase in the number of provinces located in the first and third quadrants, which indicates that the spatial agglomeration of provinces and municipalities with high and low dietary diversity has increased. There are also still more provinces and municipalities in the second and fourth quadrants, indicating that the imbalance in the level of dietary diversity between these and neighboring provinces and municipalities has not improved during the sample period.

The scatter plot of Moran Index based on the economic distance matrix shows that most provinces and cities are distributed in the third and fourth quadrants. It indicates that the dietary diversity of residents shows low-low aggregation and high-low aggregation. The provinces and cities distributed in the fourth quadrant are mainly Yunnan, Guangxi, Hainan and other provinces and cities, indicating that these provinces and cities have high resident dietary diversity, but the level of resident dietary diversity in provinces and cities with close economic distance is low. The reason is that although these provinces and cities have a lower level of economic development, they themselves have better production conditions, providing better conditions for dietary diversity. Comparing the distribution of Moran’s I index between 2000 and 2020, there is a significant increase in the number of provinces and municipalities located in the first and third quadrants, especially in the first quadrant, so that there is a more pronounced high-high aggregation of dietary diversity, mainly in the municipalities with a high level of economic development and the coastal provinces and municipalities in the south, due to the increase in the level of economic development in these provinces and municipalities.

### 3.3 Regional differences and sources of dietary diversity among Chinese residents

#### 3.3.1 Overall differences and sources of regional differences

In order to further study the regional differences in dietary diversity of Chinese residents and the sources of the differences, this paper uses the Dagum Gini coefficient to measure and decompose the regional differences in dietary diversity of Chinese residents, and the results are shown in Table 2. Figure 5 depicts the overall Gini coefficient of the dietary diversity of the Chinese residents and the contribution of the differences from 1995–2021. During the sample period, the overall Gini coefficient and the per capita disposable income of the residents change in the opposite direction, showing a decreasing trend, indicating that the regional differences in the dietary diversity of the Chinese residents during the sample period are decreasing while the per capita disposable income of the residents is rising. In addition, there are some differences in the sources of regional differences under different regional division standards. Under the three major regional division standards of East, Central and West, the contribution rate of intra-regional and hyper-variable density variations shows a decreasing trend, while the contribution rate of inter-regional variations shows an obvious upward trend after 2000, with the average contribution rate of variations reaching 58.16%, which has become the main source of regional variations in the dietary diversity of Chinese residents. Under the four major geographic regions, the contribution rates of inter-regional and intra-regional differences both showed a general trend of increasing and then decreasing, with an average contribution rate of 51.47 and 26.93%, respectively, and inter-regional differences are still the main source of regional differences in the dietary diversity of Chinese residents.

**Table 2:** Gini coefficient decomposition of dietary diversity of the Chinese population.

Year	Overall Gini coefficient	Differential contribution (%)					
		Three regions			The four main areas		
		Regional	Intra-regional	Hypervariable density	Regional	Intra-regional	Hypervariable density
1995	0.2712	32.80	33.43	33.77	30.23	48.17	21.60
2000	0.2224	30.99	46.51	22.50	28.58	45.97	25.45
2005	0.1822	28.51	56.35	15.14	26.56	51.52	21.93
2010	0.1512	26.66	63.41	9.93	24.54	55.16	20.30
2015	0.0851	24.84	65.79	9.37	25.06	59.90	15.04
2020	0.0741	24.40	61.98	13.61	26.90	56.88	16.23

Due to space constraints, Table 2 only shows the Gini coefficients of dietary diversity and their decomposition results for some years, and the complete results are shown in Supplementary Table S2.

Thus, regional imbalances in the level of dietary diversity among Chinese residents are mainly due to interregional differences in the economic and geographic diversity of residents’ diets. Moreover, with the passage of time, the imbalance of residents’ dietary diversity among economic regions is more serious than that among geographic regions. This is generally consistent with the conclusions of relevant studies and empirical observations of changes in residents’ diets. In the early stages, the level of economic development was low, and infrastructure construction was severely inadequate. Economic exchanges between regions were not as convenient as they are today. Due to limited food market supply and economic accessibility, residents’ diets largely depended on local production. Therefore, agricultural production conditions were a critical factor influencing regional dietary diversity. Liu et al. (27) have indicated in their research on the urban–rural differences in dietary diversity and food acquisition costs in China that higher food acquisition costs limit consumers’ ability to access a diverse range of foods. However, with the improvement of economic development and infrastructure, food circulation became more convenient, providing residents with a more diversified food supply. Moreover, residents gained sufficient economic capacity to afford more diverse foods. As a result, the limitations imposed by geographic conditions on dietary diversity were alleviated. Simultaneously, the increase in food diversity meant that residents had to spend more on food. In the presence of market supply, economic factors have become the primary determinant of the differences in dietary diversity.

#### 3.3.2 Intra-regional differences

Table 3 shows the trend of intra-regional Gini coefficients for the dietary diversity of residents in the three major regions and four major regions in the sample from 1995 to 2021. According to the figure, overall the intra-regional Gini coefficients of the regions are on a downward trend, and in recent years the intra-regional Gini coefficients have converged, with no great disparities, so that whether viewed from the perspective of geographic regions or economic regions, the intra-regional differences in the level of dietary diversity of the population in China are constantly shrinking and stabilizing over time. This is consistent with the trends observed in the previous kernel density maps.

**Table 3:** Intra-regional Gini coefficient of dietary diversity of the Chinese population.

Year	Three regions			The four regions			
	Eastern region	Central region	Western region	Southern region	Northern region	Northwestern region	Qinghai and Tibet
1995	0.2342	0.1988	0.3190	0.1909	0.2558	0.2833	0.5000
2000	0.1859	0.1499	0.2519	0.1389	0.2170	0.1903	0.3625
2005	0.1307	0.1243	0.1969	0.0995	0.1824	0.1676	0.2256
2010	0.0971	0.0823	0.1659	0.0782	0.1343	0.1286	0.2482
2015	0.0460	0.0418	0.0886	0.0492	0.0758	0.0439	0.1459
2020	0.0437	0.0410	0.0677	0.0467	0.0725	0.0414	0.0736

Due to space constraints, Table 3 only shows the Gini coefficients of dietary diversity and their decomposition results for some years, and the complete results are shown in Supplementary Table S3.

Based on regional agricultural production conditions and national agricultural policies, it is generally unfeasible to narrow the gap in dietary diversity through increased production diversity. The reduction in regional dietary diversity differences is more likely attributable to the simultaneous rise in economic development levels and the narrowing of economic disparities. The relevant research (28) on regional economic development disparities in China indicates that, overall, the regional economic development gap in China has shown a fluctuating yet diminishing trend, primarily reflected in the narrowing of disparities between provincial regions.

#### 3.3.3 Interregional differences

Table 4 shows the results of the interregional Gini coefficients for the three major regions as well as the four major regions for some years during the sample period. Overall, the interregional Gini coefficients show a decreasing trend under both regional division criteria, indicating that the interregional gap in the level of dietary diversity of Chinese residents is decreasing. In terms of the magnitude of the inter-regional Gini coefficient, under the three major economic region division criteria, the East–West dietary diversity difference is the largest, and the East-Central dietary diversity difference is the smallest, with the mean values of the inter-regional Gini coefficients of 0.2 and 0.14, respectively. Under the four major geographic region division criteria, the South-Qinghai-Tibet dietary diversity difference is the largest, and the dietary diversity difference of the South–North dietary diversity difference is the smallest, with the inter-regional Gini coefficients of 0.37 and 0.13. 0.37, and 0.13. In comparison, the dietary diversity of residents is more balanced among economic regions. By 2021, the inter-regional Gini coefficients of the three major economic regions will be relatively large in the eastern and western regions, while the inter-regional Gini coefficients of the four major geographic regions will be relatively large in Qinghai-Tibet and the other regions, indicating that there is still room for further balanced development of dietary diversity among the residents of China, with a focus on improving the dietary diversity of the residents of provinces and cities with a low level of economic development and poorer conditions for food production, such as Qinghai and Tibet.

**Table 4:** Inter-regional Gini coefficient of dietary diversity of the Chinese population.

Year	Three regions			The four regions					
	East-central	East-west	Central-west	South–north	South-northwest	South-Tibet	North-northwest	North-Tibet	Northwest-Tibet
1995	0.2487	0.3066	0.2665	0.2422	0.3391	0.6609	0.3166	0.6119	0.5630
2000	0.1976	0.2695	0.2146	0.1851	0.2679	0.5617	0.2822	0.5424	0.4298
2005	0.1559	0.2381	0.1810	0.1515	0.2477	0.4377	0.2447	0.4083	0.2801
2010	0.1214	0.2139	0.1496	0.1136	0.2301	0.3901	0.2179	0.3696	0.2434
2015	0.0811	0.1231	0.0751	0.0735	0.1111	0.2095	0.0849	0.1820	0.1394
2020	0.0817	0.1040	0.0609	0.0730	0.0906	0.1516	0.0692	0.1181	0.0848

Due to space constraints, Table 4 only shows the Gini coefficients of dietary diversity and their decomposition results for some years, and the complete results are shown in Supplementary Table S4.

## 4 Discussion

With the continuous improvement of people’s living standards, the dietary structure of Chinese residents has been upgraded, and dietary diversity has become an important aspect of people’s diversified material needs. In this paper, the level of dietary diversity of Chinese residents is measured on the basis of provincial panel data using the Shannon index as a measurement index, and on this basis, the regional characteristics, regional differences, and spatial and temporal evolution trends of dietary diversity of Chinese residents are explored. The main conclusions are as follows.

The overall dietary diversity of Chinese residents shows an upward trend from 1995 to 2021. Although the total calorie intake of Chinese residents during the sample period is on a downward trend, in terms of dietary structure, Chinese residents have become more homogeneous in terms of the types of food they consume. In particular, the decline in the share of calories provided by grains is very significant, while the share of calories provided by other types of food, such as meat, fruits, and vegetables, continues to increase. At the same time, the nutritional status of residents has improved, and the incidence of malnutrition has significantly decreased. In addition, the COVID-19 pandemic has impacted food consumption among Chinese residents, leading to a decline in dietary diversity levels in 2021.

There are obvious geographic and economic regional characteristics of the dietary diversity of the Chinese population. From the perspective of the four major physical geographic regions, the level of dietary diversity of the population ranges from high to low in the South, North, Northwest and Qinghai-Tibet, respectively. From the perspective of the three major economic zones, the level of dietary diversity of the population, from high to low, is in the east, center and west, respectively. Taken together, the higher dietary diversity in the provinces and cities is not necessarily due to higher levels of economic development, but may also be due to their own diversified production conditions.

There is a significant positive spatial correlation between the dietary diversity of Chinese residents, and the correlation gradually increases over time. The local Moran Index shows that there is obvious spatial aggregation in the dietary diversity of residents in 31 provinces and cities, with most provinces and cities showing “high-high” aggregation and “low-low” aggregation, and the phenomenon of aggregation has increased, and “low-low” aggregation may hinder the further improvement of the level of dietary diversity in these provinces and cities. The phenomenon of “low-low” aggregation may hinder the further improvement of dietary diversity in these provinces and cities.

Regional differences in the dietary diversity of Chinese residents have been shrinking over the sample period, but interregional differences have always been the main source of regional differences in the dietary diversity of Chinese residents. The results of regional differences in dietary diversity among Chinese residents measured and decomposed using the Dagum Gini coefficient show that both inter-regional and intra-regional differences show a trend of narrowing, whether in the four major geographic regions or the three major economic regions. However, the interregional Gini coefficients between the east and the west and between Qinghai and Tibet and the other regions are still on a downward trend, so there is still room for improvement in the interregional differences in the dietary diversity of the population.

Based on the above conclusions, this study offers the following policy recommendations. First, a nationwide dietary diversity monitoring system should be established, with attention paid to micro-level dietary data of individuals or groups. This system should integrate appropriate dietary structures to formulate nutrition improvement plans. Second, targeted regional policies should be implemented. For the central and western regions, where dietary diversity is relatively low, greater support for agricultural production should be provided to enhance the food supply chain systems in these areas. In addition, logistical networks in remote regions (such as Qinghai and Tibet) should be strengthened to improve the efficiency of food circulation, ensuring that more fresh food can flow into the market and increasing the variety of food choices available to local residents. For regions with higher dietary diversity, emphasis should be placed on dietary and nutrition health education to prevent issues such as overnutrition. Furthermore, food subsidies or application-based subsidies could be introduced in economically underdeveloped, non-remote areas to improve their access to diverse foods.

The novelty of this study lies in its macro-level measurement and analysis of dietary diversity and its spatiotemporal characteristics among Chinese residents. Based on macro-level data from a consistent source, we calculated comparable dietary diversity data within the country, which provides a reference for relevant authorities in formulating nutrition security policies. However, there are several limitations in this study. First, while the macro-level measurement of dietary diversity ensures comparability across regions, it can only provide a rough reflection of overall dietary diversity or that of different groups (by geographic unit), neglecting individual, family, or specific group dietary differences. Additionally, this study did not account for regional dietary cultures and preferences, which to some extent overlooked the reasonableness of lower dietary diversity. Second, this study is conducted from a national perspective, lacking a global comparative and exploratory viewpoint. Lastly, the results of this study can serve as a benchmark for designing nutrition intervention strategies for communities or government organizations. However, as previously mentioned, higher dietary diversity is not always better, and the specific dietary needs of different groups should be specially considered. Future research should focus on the dietary diversity of different groups and its nutritional health rationality, or explore dietary diversity and regional differences in a globally comparable context.

## Data Availability

The data analyzed in this study is subject to the following licenses/restrictions: the datasets presented in this article are not readily available because this study is ongoing. However, the measurement data used to support the results of this study are available from the corresponding author upon reasonable request. Requests to access these datasets should be directed to Shiwen Quan, quanshiwen@163.com.

## Footnotes

^1^This article simultaneously measured the Shannon index and Simpson index during actual data processing, and found a strong correlation between the two. There was no significant difference in information when measuring the level of dietary diversity in this article, so the Shannon index was chosen as the main measure.

^2^Data sourced from the "China National Nutrition and Chronic Disease Report (2015)".
