# Supplementary material for: Measurement and spatiotemporal evolution characteristics of dietary diversity among Chinese residents
Source: Front Nutr. 2025 Jan 27;12:1480133. doi: 10.3389/fnut.2025.1480133 (PMC11807821; doi:10.3389/fnut.2025.1480133)
Supplement: Supplementary file 1 [file Table_1.DOCX]

Supplementary Material

Table 1: Criteria for regionalization

| By physical geography | |
| --- | --- |
| Northern region | Beijing, Jilin, Tianjin, Shandong, Shanxi, Hebei, Henan, Liaoning, Shaanxi, Heilongjiang |
| Southern region | Shanghai, Yunnan, Sichuan, Anhui, Guangdong, Guangxi, Jiangsu, Jiangxi, Zhejiang, Hainan,  Hubei, Hunan, Fujian, Guizhou, Chongqing |
| Northwest region | Inner Mongolia, Ningxia, Xinjiang, Gansu |
| Qinghai and Tibet | Qinghai, Tibet |
| By economic development | |
| eastern region | Beijing, Tianjin, Hebei, Liaoning, Shanghai, Shandong, Zhejiang, Fujian, Guangdong, Hainan,  Jiangsu |
| central region | Shanxi, Jilin, Heilongjiang, Anhui, Jiangxi, Henan, Hubei, Hunan, |
| western region | Inner Mongolia, Guangxi, Chongqing, Sichuan, Guizhou, Yunnan, Tibet, Shaanxi, Gansu, Qinghai,  Ningxia, Xinjiang |

Table 2: Gini coefficient decomposition of dietary diversity of the Chinese population

| vintages | Overall Gini coefficient | Differential contribution (%) | | | | | |
| --- | --- | --- | --- | --- | --- | --- | --- |
|  |  | three regions | | | four areas | | |
|  |  | regional | intra-regional | hypervariable density | regional | intra-regional | hypervariable density |
| 1995 | 0.2712 | 32.80 | 33.43 | 33.77 | 30.23 | 48.17 | 21.60 |
| 1996 | 0.2492 | 32.64 | 31.67 | 35.69 | 29.84 | 45.02 | 25.14 |
| 1997 | 0.2407 | 32.55 | 32.26 | 35.19 | 30.48 | 41.47 | 28.05 |
| 1998 | 0.2280 | 32.78 | 29.75 | 37.47 | 30.39 | 41.29 | 28.33 |
| 1999 | 0.2190 | 32.83 | 29.54 | 37.63 | 30.31 | 40.54 | 29.15 |
| 2000 | 0.2224 | 30.99 | 46.51 | 22.50 | 28.58 | 45.97 | 25.45 |
| 2001 | 0.2113 | 31.69 | 40.82 | 27.49 | 28.38 | 46.31 | 25.31 |
| 2002 | 0.1912 | 31.62 | 42.32 | 26.06 | 28.55 | 45.15 | 26.30 |
| 2003 | 0.1843 | 30.90 | 47.35 | 21.75 | 27.93 | 48.58 | 23.49 |
| 2004 | 0.1955 | 30.88 | 45.60 | 23.52 | 27.92 | 46.36 | 25.72 |
| 2005 | 0.1822 | 28.51 | 56.35 | 15.14 | 26.56 | 51.52 | 21.93 |
| 2006 | 0.1723 | 28.34 | 58.22 | 13.44 | 26.75 | 49.68 | 23.57 |
| 2007 | 0.1675 | 28.85 | 56.50 | 14.66 | 26.32 | 48.40 | 25.28 |
| 2008 | 0.1614 | 28.18 | 59.06 | 12.75 | 24.96 | 52.99 | 22.05 |
| 2009 | 0.1497 | 27.90 | 61.37 | 10.73 | 25.22 | 49.06 | 25.72 |
| 2010 | 0.1512 | 26.66 | 63.41 | 9.93 | 24.54 | 55.16 | 20.30 |
| 2011 | 0.1442 | 26.58 | 62.72 | 10.70 | 23.74 | 59.01 | 17.25 |
| 2012 | 0.1411 | 26.27 | 63.97 | 9.76 | 23.43 | 59.69 | 16.88 |
| 2013 | 0.1203 | 25.67 | 64.98 | 9.35 | 23.80 | 58.80 | 17.40 |
| 2014 | 0.0972 | 26.35 | 64.15 | 9.50 | 24.56 | 57.41 | 18.03 |
| 2015 | 0.0851 | 24.84 | 65.79 | 9.37 | 25.06 | 59.90 | 15.04 |
| 2016 | 0.0808 | 24.78 | 64.68 | 10.54 | 25.92 | 57.77 | 16.32 |
| 2017 | 0.0810 | 24.94 | 65.06 | 10.00 | 25.31 | 58.04 | 16.64 |
| 2018 | 0.0727 | 24.76 | 64.38 | 10.86 | 25.89 | 57.34 | 16.77 |
| 2019 | 0.0706 | 24.95 | 61.65 | 13.40 | 26.73 | 56.51 | 16.76 |
| 2020 | 0.0741 | 24.40 | 61.98 | 13.61 | 26.90 | 56.88 | 16.23 |
| 2021 | 0.0624 | 24.68 | 62.66 | 12.66 | 28.82 | 52.64 | 18.54 |

Table 3: Intra-regional Gini coefficient of dietary diversity of the Chinese population

| vintages | three regions | | | the four regions | | | |
| --- | --- | --- | --- | --- | --- | --- | --- |
|  | eastern region | central region | western region | southern region | northern region | northwest region | Qinghai and Tibet |
| 1995 | 0.2342 | 0.1988 | 0.3190 | 0.1909 | 0.2558 | 0.2833 | 0.5000 |
| 1996 | 0.2269 | 0.1805 | 0.2787 | 0.1682 | 0.2383 | 0.2776 | 0.1010 |
| 1997 | 0.2341 | 0.1641 | 0.2576 | 0.1756 | 0.2142 | 0.2595 | 0.0916 |
| 1998 | 0.2259 | 0.1604 | 0.2398 | 0.1683 | 0.2000 | 0.2238 | 0.1744 |
| 1999 | 0.2148 | 0.1619 | 0.2286 | 0.1558 | 0.2050 | 0.2323 | 0.1873 |
| 2000 | 0.1859 | 0.1499 | 0.2519 | 0.1389 | 0.2170 | 0.1903 | 0.3625 |
| 2001 | 0.1891 | 0.1445 | 0.2357 | 0.1271 | 0.2167 | 0.1880 | 0.3247 |
| 2002 | 0.1781 | 0.1313 | 0.2025 | 0.1170 | 0.1968 | 0.1780 | 0.2543 |
| 2003 | 0.1546 | 0.1412 | 0.1967 | 0.1095 | 0.1887 | 0.1527 | 0.3275 |
| 2004 | 0.1634 | 0.1502 | 0.2087 | 0.1135 | 0.2061 | 0.1663 | 0.2776 |
| 2005 | 0.1307 | 0.1243 | 0.1969 | 0.0995 | 0.1824 | 0.1676 | 0.2256 |
| 2006 | 0.1234 | 0.1081 | 0.1885 | 0.0977 | 0.1661 | 0.1636 | 0.2412 |
| 2007 | 0.1140 | 0.1114 | 0.1942 | 0.0891 | 0.1657 | 0.1856 | 0.3138 |
| 2008 | 0.1075 | 0.0928 | 0.1901 | 0.0811 | 0.1506 | 0.1794 | 0.2689 |
| 2009 | 0.1001 | 0.0853 | 0.1731 | 0.0782 | 0.1353 | 0.1705 | 0.2992 |
| 2010 | 0.0971 | 0.0823 | 0.1659 | 0.0782 | 0.1343 | 0.1286 | 0.2482 |
| 2011 | 0.0888 | 0.0746 | 0.1635 | 0.0699 | 0.1314 | 0.1002 | 0.2959 |
| 2012 | 0.0874 | 0.0634 | 0.1608 | 0.0690 | 0.1235 | 0.0847 | 0.3368 |
| 2013 | 0.0748 | 0.0472 | 0.1323 | 0.0608 | 0.1079 | 0.0697 | 0.2384 |
| 2014 | 0.0589 | 0.0381 | 0.1111 | 0.0523 | 0.0876 | 0.0652 | 0.1904 |
| 2015 | 0.0460 | 0.0418 | 0.0886 | 0.0492 | 0.0758 | 0.0439 | 0.1459 |
| 2016 | 0.0423 | 0.0464 | 0.0813 | 0.0478 | 0.0764 | 0.0465 | 0.1214 |
| 2017 | 0.0441 | 0.0439 | 0.0822 | 0.0459 | 0.0765 | 0.0433 | 0.1415 |
| 2018 | 0.0400 | 0.0412 | 0.0711 | 0.0429 | 0.0700 | 0.0405 | 0.0833 |
| 2019 | 0.0429 | 0.0395 | 0.0658 | 0.0438 | 0.0691 | 0.0439 | 0.0661 |
| 2020 | 0.0437 | 0.0410 | 0.0677 | 0.0467 | 0.0725 | 0.0414 | 0.0736 |
| 2021 | 0.0401 | 0.0313 | 0.0563 | 0.0451 | 0.0601 | 0.0352 | 0.0485 |

Table 4: Inter-regional Gini coefficient of dietary diversity of the Chinese population

| vintages | three regions | | | the four regions | | | | | |
| --- | --- | --- | --- | --- | --- | --- | --- | --- | --- |
|  | east-central | east-west | central-west | south-north | south-northwest | south-Tibet | north-northwest | north-Tibet | northwest-Tibet |
| 1995 | 0.2487 | 0.3066 | 0.2665 | 0.2422 | 0.3391 | 0.6609 | 0.3166 | 0.6119 | 0.5630 |
| 1996 | 0.2335 | 0.2819 | 0.2364 | 0.2195 | 0.3160 | 0.5318 | 0.3061 | 0.4849 | 0.4335 |
| 1997 | 0.2268 | 0.2777 | 0.2197 | 0.2023 | 0.3019 | 0.5228 | 0.2985 | 0.5004 | 0.4276 |
| 1998 | 0.2161 | 0.2592 | 0.2101 | 0.1936 | 0.2766 | 0.5004 | 0.2717 | 0.4829 | 0.4063 |
| 1999 | 0.2096 | 0.2485 | 0.2002 | 0.1882 | 0.2493 | 0.4927 | 0.2541 | 0.4723 | 0.4288 |
| 2000 | 0.1976 | 0.2695 | 0.2146 | 0.1851 | 0.2679 | 0.5617 | 0.2822 | 0.5424 | 0.4298 |
| 2001 | 0.1846 | 0.2493 | 0.2100 | 0.1808 | 0.2530 | 0.5032 | 0.2714 | 0.4833 | 0.3750 |
| 2002 | 0.1708 | 0.2277 | 0.1834 | 0.1673 | 0.2268 | 0.4229 | 0.2428 | 0.4032 | 0.3040 |
| 2003 | 0.1661 | 0.2197 | 0.1821 | 0.1618 | 0.2185 | 0.4356 | 0.2175 | 0.4117 | 0.3187 |
| 2004 | 0.1778 | 0.2317 | 0.1939 | 0.1763 | 0.2367 | 0.4313 | 0.2422 | 0.4070 | 0.2981 |
| 2005 | 0.1559 | 0.2381 | 0.1810 | 0.1515 | 0.2477 | 0.4377 | 0.2447 | 0.4083 | 0.2801 |
| 2006 | 0.1457 | 0.2288 | 0.1689 | 0.1385 | 0.2314 | 0.4189 | 0.2342 | 0.4003 | 0.2791 |
| 2007 | 0.1368 | 0.2179 | 0.1724 | 0.1357 | 0.2161 | 0.4043 | 0.2318 | 0.4001 | 0.3084 |
| 2008 | 0.1250 | 0.2187 | 0.1662 | 0.1270 | 0.2276 | 0.4141 | 0.2280 | 0.3905 | 0.2898 |
| 2009 | 0.1120 | 0.2066 | 0.1554 | 0.1136 | 0.1967 | 0.4021 | 0.2080 | 0.3940 | 0.3084 |
| 2010 | 0.1214 | 0.2139 | 0.1496 | 0.1136 | 0.2301 | 0.3901 | 0.2179 | 0.3696 | 0.2434 |
| 2011 | 0.1149 | 0.2039 | 0.1448 | 0.1093 | 0.2159 | 0.3939 | 0.1949 | 0.3682 | 0.2709 |
| 2012 | 0.1128 | 0.2026 | 0.1388 | 0.1054 | 0.2096 | 0.4051 | 0.1837 | 0.3791 | 0.2843 |
| 2013 | 0.1014 | 0.1748 | 0.1136 | 0.0942 | 0.1732 | 0.3232 | 0.1481 | 0.2981 | 0.2130 |
| 2014 | 0.0853 | 0.1392 | 0.0875 | 0.0825 | 0.1215 | 0.2552 | 0.1006 | 0.2303 | 0.1854 |
| 2015 | 0.0811 | 0.1231 | 0.0751 | 0.0735 | 0.1111 | 0.2095 | 0.0849 | 0.1820 | 0.1394 |
| 2016 | 0.0815 | 0.1146 | 0.0702 | 0.0725 | 0.0974 | 0.1926 | 0.0783 | 0.1658 | 0.1266 |
| 2017 | 0.0812 | 0.1155 | 0.0695 | 0.0720 | 0.0961 | 0.2038 | 0.0768 | 0.1788 | 0.1403 |
| 2018 | 0.0702 | 0.1037 | 0.0657 | 0.0660 | 0.0900 | 0.1673 | 0.0719 | 0.1408 | 0.0984 |
| 2019 | 0.0738 | 0.0988 | 0.0604 | 0.0681 | 0.0886 | 0.1438 | 0.0695 | 0.1131 | 0.0774 |
| 2020 | 0.0817 | 0.1040 | 0.0609 | 0.0730 | 0.0906 | 0.1516 | 0.0692 | 0.1181 | 0.0848 |
| 2021 | 0.0665 | 0.0891 | 0.0506 | 0.0620 | 0.0666 | 0.1270 | 0.0535 | 0.0982 | 0.0755 |
